# The Role of Purinergic Mechanisms in the Excitability of Trigeminal Afferents of Rats with Prenatal Hyperhomocysteinemia

**DOI:** 10.3390/biom15030419

**Published:** 2025-03-15

**Authors:** Elizaveta Ermakova, Svetlana Svitko, Alsu Kabirova, Egor Nevsky, Olga Yakovleva, Karina Gilizhdinova, Kseniia Shaidullova, Anton Hermann, Guzel Sitdikova

**Affiliations:** 1 Department of Human and Animal Physiology, Institute of Fundamental Medicine and Biology, Kazan Federal University, 18 Kremlevskaya Str., 420008 Kazan, Russia; elivermakova@kpfu.ru (E.E.); sveosvitko@kpfu.ru (S.S.); alsuakabirova@kpfu.ru (A.K.); egsnevskiy@kpfu.ru (E.N.); olga.jakovleva@kpfu.ru (O.Y.); karrgilizhdinova@kpfu.ru (K.G.); kskoroleva@kpfu.ru (K.S.); 2 Department of Biosciences, University of Salzburg, Hellbrunnerstr. 34, 5020 Salzburg, Austria; anton.hermann@plus.ac.at

**Keywords:** migraine, homocysteine, trigeminal ganglion (TG) cells, meningeal afferents, mast cells, calcitonin gene-related peptide (CGRP), purinergic receptors

## Abstract

Elevated levels of homocysteine in the blood plasma (hyperhomocysteinemia, HHCY) positively correlate with migraine symptoms in patients. Experimental studies show a higher sensitivity of rats with prenatal HHCY (pHHCY) to migraine symptoms like allodynia, photophobia, anxiety, and a higher excitability of meningeal trigeminal afferents. In the present study, the roles of purinergic mechanisms in the homocysteine-induced hyperexcitability of the trigeminal ganglion (TG) system using electrophysiological recordings from the trigeminal nerve, Ca^2+^ imaging of cells isolated from TG, and mast cell staining in meninges were investigated. Experiments were performed using rats with pHHCY born from females fed with a high-methionine-containing diet before and during pregnancy. Firstly, we found that lower concentrations of 4-aminopyridine, a K^+^-channel blocker, were able to induce an increase in the nociceptive activity of trigeminal afferents, supporting the hypothesis of the higher excitability of the trigeminal nerve of rats with pHHCY. Trigeminal afferents of rats with pHHCY were more sensitive to the exogenous application of the nonspecific agonist of purinergic ATP receptors. In neurons and satellite glial cells of TG of rats with pHHCY ATP, ADP (an agonist of metabotropic P2Y receptors) and BzATP (an agonist of ionotropic P2X with especially high potency for the P2X7 receptor) induced larger Ca^2+^ transients. The incubation of TG neurons in homocysteine for 24 h increased the ratio of neurons responding simultaneously to ATP and capsaicin. Moreover, rats with pHHCY exhibit a higher rate of degranulation of mast cells and increased response to the agonist of the P2X7 receptor BzATP application. In addition, higher levels of calcitonin gene-related peptide (CGRP) were found in rats with pHHCY. Our results suggest that chronic elevated levels of homocysteine induce the upregulation of ionotropic or metabotropic ATP receptors in neurons, satellite glial cells, and mast cells, which further provide inflammatory conditions and the sensitization of peripheral afferents underlying pain.

## 1. Introduction

Homocysteine is a sulfur-containing amino acid produced during a cascade of reactions from methionine consumed with food [1,2,3,4]. Methionine is converted to S-adenosylmethionine (SAM) by methionine adenosyltransferase. SAM serves as a methyl donor in various methyltransferase reactions. Glycine N-methyltransferase converts SAM to S-adenosylhomocysteine (SAH), which further undergoes reversible hydrolysis by adenosyl-homocysteine hydrolase, yielding homocysteine. Homocysteine can be remethylated into methionine in the methionine synthase reaction, where the one-carbon methyl group of methylenetetrahydrofolate (5-methylene, THF) is transferred to homocysteine. The cofactor for this reaction is vitamin B12 in the form of methylcobalamin. In the transsulfuration pathway, homocysteine is converted to cysteine by the enzyme cystathionine-β-synthase using B12 as a co-factor [3,4,5]. An increase in the concentration of homocysteine in blood plasma above 15 μM is considered as hyperhomocysteinemia (HHCY) [5]. HHCY can develop as a result of genetic mutations of the methionine cycle enzymes (the cause of the most severe cases of HHCY), including 5,10-methylenetetrahydrofolate reductase (MTHFR) polymorphism. Also, HHCY can be a result of kidney disease; dietary habits; taking certain medications, like L-DOPA or antiepileptic drugs; aging; smoking; alcohol consumption; and a sedentary lifestyle [6,7,8,9,10,11]. HHCY conditions can be modeled in rodents with high methionine or/and vitamin B-deficient diets, as well as with acute chronic homocysteine injections or using genetic mutations of CBS or MTHFR [3,12]. Maternal HHCY associated with elevated homocysteine levels in dams during pregnancy induces not only well-known complication such as fetal growth restriction, preeclampsia, preterm birth but also a delay of the sensorimotor development of the offspring in the early neonatal period and permanent cognitive and motor deficits in postnatal life [13,14,15,16,17].

Clinical data indicate an increase in the frequency and severity of migraines (especially migraine attacks with aura) in patients with elevated plasma or CSF homocysteine levels [18,19,20,21,22]. Our previous studies indicate that rats with prenatal HHCY (pHHCY) exhibit mechanical allodynia, photophobia, anxiety, and a decrease in the thresholds for the generation of cortical spreading depression, an electrophysiological correlate of aura [23]. Moreover, rats with pHHCY were more sensitive in the model of chronic nitroglycerine-induced migraines [24]. These data suggest the sensitization of both central and peripheral structures responsible for migraine development and propose homocysteine as a migraine biomarker [25].

The mechanisms contributing to the pain phase of migraines require the activation of trigeminal afferents and the transmission of nociceptive information from the cranial meninges into the central nervous system in a region of the dorsal brainstem known as the trigeminal nucleus caudalis [26]. Most meningeal afferents are characterized by polymodal sensitivity to chemical, mechanical, and thermal stimulation and have slow conduction velocities in the A-delta and C-fiber ranges [27,28,29,30]. Accordingly, the purinergic mechanisms of migraine extracellular ATP is one of the key players in pain generation and the sensitization of primary sensory neurons and their afferents [31].

Ionotropic and metabotropic ATP receptors are expressed in trigeminal ganglion (TG) neurons and their peripheral afferents, satellite glial cells (SGCs), and mast cells [31]. ATP triggers nociceptive firing in trigeminal nerve fibers present in whole-mount rat meninges [32] and induces mast cell degranulation with the further release of 5-hydroxytryptamine (5-HT) which can not only induce [33] the nociceptive firing of meningeal afferents [34] but also provide further sensitization of afferents [35]. The major migraine mediator calcitonin gene-related peptide (CGRP) is known to sensitize P2X3 receptors to increase impulse transmission to brainstem trigeminal nuclei [36].

Neuroinflammation leads to increased permeability of the blood–brain barrier and blood vessels, and ATP is highly involved in the massive spread of the inflammatory reaction [37,38]. Inflammation and a long-term sensitization of nociceptive neurons cause an increase in the activity and expression of P2X3 receptors, which play a major role in the occurrence of chronic migraines [31,39]. A number of studies demonstrate that chronic HHCY induces oxidative stress and increases the levels of proinflammatory cytokines like interleukins IL-6 and IL-1β, as well as TNF-α in the cerebral cortex and plasma [40,41,42,43]. Homocysteine is easily oxidized in the presence of oxygen with the formation of disulfides and reactive oxygen species like superoxide anion, hydrogen peroxide, or hydroxyl radicals. In addition, the suppression of the activity, translation, and transcription of antioxidant enzymes, such as superoxide dismutase (SOD), catalase (CAT), and glutathione peroxidase (GPx), and the simultaneous activation of NADPH oxidases was shown in HHCY [44,45,46]. One of the suggested targets of homocysteine is toll-like receptor 4 (TLR-4), and its mutation can attenuate the effects of HHCY-mediated vascular inflammation and mitochondria-dependent cell death. TLRs are expressed on immune (macrophages, dendritic cells, and monocytes) as well as non-immune cells (endothelial cells, smooth muscle cells, fibroblasts, and epithelial cells), and its activation promotes inflammatory cytokine up-regulation [47]. In addition, the activation of NF-kB, an inflammatory cytokine transcription factor, was observed in HHCY [48,49,50]. In the model of pHHCY, the increased levels of IL-1β, TNF-α, IL-6, and oxidative markers were observed in plasma and brain tissues [17,51]. These proinflammatory molecules can directly pass from the blood circulation into the dura mater and induce local inflammation [52]. The high rate of mast cell degranulation revealed in the meninges of rats with pHHCY promotes the development of “neurogenic inflammation” during chronic increases in the homocysteine level [53,54,55].

Therefore, it was proposed that inflammatory conditions on pHHCY promote the sensitization of the peripheral afferents, followed by increased excitability and persistent neuronal discharge, which could drive an ongoing migraine headache [27,52].

However, there is no experimental data indicating changes in the sensitivity of the trigeminal nerve and TG cells to ATP in the conditions of HHCY. In the present study, using the pHHCY rat model, we analyzed (i) the sensitivity of trigeminal afferents to excitatory effects of 4-aminopyridine (4-AP), (ii) the ATP-evoked electrical activity in the trigeminal nerve, (iii) the changes in cytosolic Ca^2+^ levels in the cells isolated from the trigeminal ganglion in response to agonists of ATP receptors, (iv) the rate of mast cell degranulation induced by the P2X7 receptor agonist, and (v) the levels of CGRP in blood plasma.

## 2. Materials and Methods

### 2.1. Experimental Animals: The Model of Prenatal HHCY

The experiments were carried out in accordance with the EU Directive 2010/63 / EU for animal experiments and approved by the Local Ethics Committee of the Kazan Federal University (protocols N. 8 dated 5 May 2015, N. 33 dated 25 November 2021). Wistar rats were housed in cages under a 12:12 h light/dark cycle (the light was switched on at 6 AM every day) at a temperature of 22 °C and in a humidity-controlled room with food and water ad libitum. Experimental animals were divided into 2 groups: Rats from the control group were born from females fed with a standard diet (n = 12). Rats from the pHHCY group were born from females (n = 10) that received a high-methionine-containing diet daily (7.7 g/kg meal) three weeks before and during pregnancy [3,14,17,51,56].

### 2.2. Plasma Homocysteine and CGRP Concentration

Blood sampling in rats was performed using gum incision. The samples were then mixed with an anticoagulant in tubes and centrifuged at 1500 rpm for 15 min. The supernatant was separated and stored at −40 °C until ELISA analysis.

Plasma homocysteine levels were measured in female rats and their offspring using a homocysteine colorimetric assay kit (E-BC-K143, ElabScience, Waltham, MA, USA) via spectrophotometry using an ELISA reader (Multiskan FS, Thermo Fisher Scientific, Waltham, MA, USA). The method is based on the following reactions: Oxidized homocysteine is reduced to free homocysteine by triethylphosphine (TCEP), and the free homocysteine reacts with the substrate to generate adenosine. Adenosine is immediately dehydrogenated into inosine and ammonia, and ammonia further reacts with NADH under the catalysis of glutamate dehydrogenase to convert NADH to NAD+. The decrease in absorbance at 340 nm caused by the decline of NADH is proportional to the concentration of homocysteine in the sample, measured in μM/L.

The concentrations of CGRP were determined using rat-specific ELISA kits (CEA876Ra ELISA Kit for Calcitonin Gene Related Peptide, CGRP, Cloud-Clone Corp., Katy, TX, USA) according to the manufacturer’s instructions, and the samples were added to the corresponding micro-ElISA strip-plate wells. Specific horseradish peroxidase (HRP)-conjugated antibodies were added to each sample for incubation, followed by chromogen solutions A and B for coloring, and finally, a stop solution was added to terminate the reaction, changing the visible color from blue to yellow. The amount of bound HRP conjugates and the intensity of the color are inversely proportional to the concentration of CGRP in the sample. Optical density (OD) was calculated using a microplate spectrophotometer (Multiskan FS, Thermo Fisher Scientific, Waltham, MA, USA) at a wavelength of 450 nm. Each sample was analyzed through a calibration curve, and the mean concentrations were calculated and expressed as pg/mL.

### 2.3. Electrophysiological Recordings of the Electrical Activity of Trigeminal Afferents in Hemiskull Preparations

The activity of trigeminal afferents was recorded using isolated rat hemiskulls with intact dural innervation obtained from rats P40–45, as was described previously [55,57,58]. The rat skull after decapitation was carefully cleaned of all cranial muscles; then, it was divided along the sagittal section into two hemiskulls, and the brain was removed from the cranium. The distal part of the nervus spinosis that innervates the receptive field around the middle meningeal artery was isolated from the hemiskull and placed inside the recording electrode. Male rats were used in our study to avoid any instability induced by hormonal changes during the estrous cycle in females. The preparations were perfused with an artificial cerebrospinal fluid containing 120 mM NaCl, 2.5 mM KCl, 2 mM CaCl_2_, 1 mM MgCl_2_, 11 mM glucose, 1 mM NaHPO_4_, and 24 mM NaHCO_3_ under constant oxygenation at 95% O_2_/CO_2_, a pH range of 7.2–7.35, and at 20–25 °C [58]. The action potentials (APs) of the trigeminal nerve were recorded using a DAM 80 amplifier and digitized on a PC with an NI PCI6221 board (National Instruments, Austin, TX, USA). The signals were visualized and analyzed using WinEDR v.3.2.7 software (University of Strathclyde, Glasgow, UK). The AP frequency was measured and analyzed using the DoClust application in the MATLABver. 9.8 (R2020a) package (MathWorks, Natick, MA, USA) and OriginPro 2015 (OriginLab Corporation, Northampton, MA, USA) software.

### 2.4. Ca^2+^ Imaging in the Culture of the Trigeminal Ganglion

The primary culture of TG cells (neurons and glial cells) was prepared from the TG of P8-12 rats as described previously [57,58]. The TG cells were removed after rat decapitation and placed in a cold F12 medium. After that, TG cells were dissociated using an enzymatic cocktail containing 0.25 mg/mL trypsin, 1 mg/mL collagenase, and 0.2 mg/mL DNAse in shaker at a temperature of 37 °C for 25 min. Dissociated cells were placed on coverslips coated with poly-L-lysine for 24 h before experiments.

To image Ca^2+^ cells, Fluo4-AM (2 μM, Invitrogen) was used as described previously [55,58]. To detect and measure changes in the intracellular calcium levels of cultured TG cells, they were stained using Fluo4-AM in the presence of pluoronic acid (0.02%) at 37 °C for 30–40 min in the dark, followed by washout with an extracellular solution for 10 min. Fluorescence visualization of stained cells was performed using an Axio observer D1 microscope (Carl Zeiss, Oberkochen, Germany) with an excitation filter (BP 450–490 nm), a beam splitter (FT 510 nm), and an emission filter (LP 555 nm). Fluorescence images were recorded using an AxioCam MRm high-speed camera (Carl Zeiss, Oberkochen, Germany). All substances were applied using a gravity-controlled perfusion system (ALA Scientific Instruments Westbury, Farmingdale, NY, USA). At the end of each experiment, KCl (50 mM, 2 s) was applied to differentiate neuronal and glial cells. Fluorescence images were converted into signals using ImageJ1.52s (NIH, Bethesda, MD, USA), and fluorescence intensity was estimated in arbitrary units (a.u.). The peak fluorescence was expressed as the relative change in fluorescence (F − F_0_)/F_0_ for each frame of each measurement, where F is the peak fluorescence of the cell and F_0_ is the background fluorescence close to a given cell. Any changes in fluorescence intensity that were beyond the flat baseline at the time of the agonist application and had a characteristic asymmetric shape with a rapid rise and a slow decline were considered as Ca^2+^ responses. Full width at half maximum (FWHM) was determined as the difference between the time taken at the level of half the amplitude of the Ca^2+^ response and represented the duration of the signal. The fluorescent response to KCl was used to normalize amplitudes of the responses of TG neurons for all substances used in the current study. The MATLABver. 9.8 (R2020a) (The MathWorks, Natick, MA, USA) software package and OriginPro 2015 (OriginLab Corporation, Northampton, MA, USA) were used for quantitative and statistical analysis of evoked Ca^2+^ responses.

### 2.5. Toluidine Blue Staining of Meningeal Mast Cells

The rate of mast cell degranulation was studied using toluidine blue (Sigma-AldrichGmbH, Schnelldorf, Germany); the staining of the meninges of P35–45 rats were obtained from isolated hemiskulls, as described previously [59,60,61]. After removing the external cranial muscles, the skulls were divided by a sagittal incision into two halves, and the brain was extracted. The preparation was placed in an artificial cerebrospinal fluid solution (control or containing BzATP according to the protocol) for 30 min and then fixed in paraformaldehyde (4%) overnight at 4 °C. Before the isolation of the meninges, the samples were washed in a phosphate-buffered solution containing (in mM) 137 NaCl, 2.7 KCl, 10 Na2HPO4, and 1.8 KH2PO4. Isolated meninges were stained with toluidine blue for 10 min and then washed with distilled water and dehydrated with ethanol (95% and 99%). Images were visualized with an Olympus AX-TFSM microscope (Olympus, Hachioji, Tokyo, Japan) at 20× magnification. Mast cell degranulation was evaluated visually in a blind manner by analyzing 100 cells from 10 different fields of view in each preparation. The rate of degranulation was calculated as the ratio of degranulated cells to the total number of studied cells.

### 2.6. Chemicals

The following chemicals were used: 4-aminopiridine (4-AP), ATP, ADP, BzATP, and L-homocysteine. All chemicals were from Sigma-Aldrich (Sigma–Aldrich GmbH, Schnelldorf, Germany).

### 2.7. Statistical Analysis

Data analysis was performed using the MATLAB package (MathWorks, Natick, MA, USA) and Origin Pro 2015 (OriginLab Corporation, Northampton, USA). EC50 was calculated from dose-dependence curves using sigmoidal curve-fitting (y = A1 + (A2 − A1)/(1 + 10^(Logx0−x)p^), where A1 is the minimum effect, A2 is the maximum effect, Logx0 is the center (10Logx0  =  EC50), and p is the power of the sigmoidal curve) [62]. The Shapiro–Wilk test was used to determine the normality of the dataset. All hypothesis testing was two-tailed. The nonparametric Mann–Whitney U-test or Student’s t-test was used to compare the two independent groups. The two-sided Wilcoxon rank sum test for paired samples or the paired t-test was used to assess the effect of the compounds in the same preparation. The chi-square test was used for Ca^2+^ imaging data to compare the percentage of the responding cells in the different groups. Differences were considered statistically significant at *p* < 0.05; n represents the number of animals.

## 3. Results

### 3.1. Plasma Homocysteine and CGRP Levels

Plasma homocysteine levels were 17.3 ± 2.3 μM (n = 10) in females on a methionine diet compared to 6.3 ± 1.0 μM (n = 12) in control female rats. The homocysteine level in the rat pups of the control group was 7.4 ± 1.2 μM (n = 12), and 16.1 ± 0.9 μM (n = 10 Mann–Whitney test, *p* = 0.000186) in the group with pHHCY. The CGRP plasma level was 25.8 ± 1.9 pg/mL (n = 10) in the control group and 34.2 ± 3.2 pg/mL (n = 9, Mann–Whitney test, *p* = 0.037) in rats with pHHCY.

### 3.2. 4-Aminopyridine Increases the Frequency of Action Potentials of Trigeminal Afferents in Rats with pHHCY

**Figure 1:**
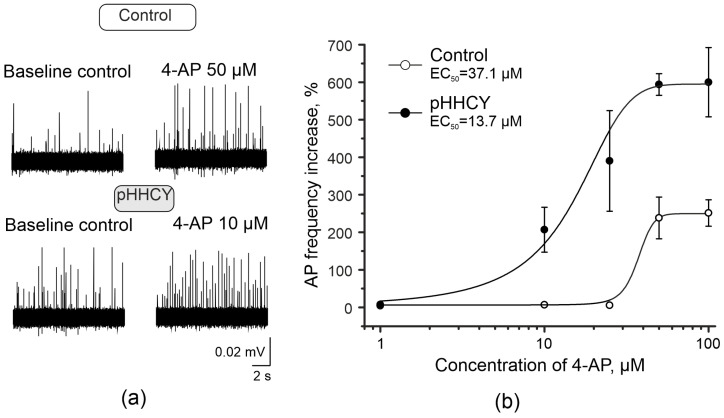
The electrical activity of the trigeminal nerve changes in response to 4-aminopyridine (4-AP) application. (**a**) Examples of AP recordings from the trigeminal nerve of control rats and pHHCY rats before and after 4-AP application; (**b**) Dose-dependent effects of 4-AP (1–100 μM) on the average number of APs in 20 min in the trigeminal nerve. The effective concentration (EC50) of 4-AP on the AP frequency in the control group was 37 μM; in the pHHCY group, it was 14 μM. The Y-axis shows the increase in the AP frequency in 20 min (%); the X- axis shows the concentration of 4-AP in μM.

To analyze the excitability of trigeminal afferents, we applied 4-AP, a blocker of voltage-gated potassium channels (Figure 1a), into the receptive area of the dura mater. We tested the following concentrations: 10 μM, 25 μM, 50 μM, and 100 μM. In rats from the control group, the minimum concentration of 4-AP that significantly increased the AP frequency was 50 μM (from 939.6 ± 105.2 APs in 20 min to 3469.1 ± 820.1 APs in 20 min; n = 8; Wilcoxon signed-rank test, *p* = 0.014; Table 1). The effective concentration (EC50) of 4-AP on the AP frequency in the control group was 37 μM (Figure 1b). In the pHHCY group, the threshold concentration of 4-AP that significantly increased the AP frequency was 10 μM: frequency changed from 1282.5 ± 75.7 APs in 20 min before to 3813.1 ± 586.3 APs in 20 min after 4-AP application (n = 6; Wilcoxon signed-rank test, *p* = 0.036; Figure 1b; Table 1). The EC50 of 4-AP on the AP frequency in the pHHCY group is 14 μM (Figure 1b).

### 3.3. Effects of ATP on the Electrical Activity of the Trigeminal Nerve in Rats with pHHCY

**Figure 2:**
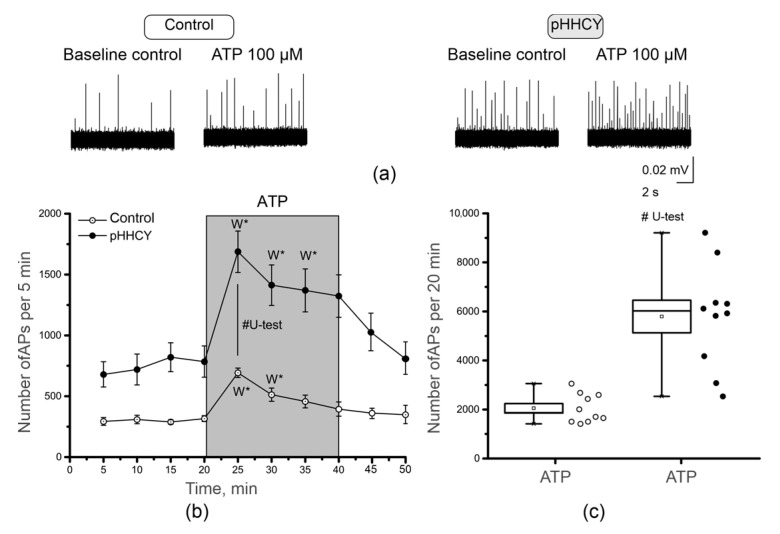
The effects of ATP on the electrical activity of trigeminal afferents. (**a**) Original recordings of APs from the trigeminal nerve of rats from the control and pHHCY groups before and after the application of ATP (100 μM); (**b**) the time course of the frequency of APs (number per 5 min) before and after the application of ATP (100 μM) in the control (n = 10) and pHHCY (n = 10) groups. Data are presented as mean ± SEM; (**c**) a boxplot representing the average number of APs per 20 min during ATP application in the control and pHHCY groups. Data are presented as a box (SEM); the line is the median; the white square in the box is the mean value; whiskers are the minimum and maximum values; white and black dots are the original data. * *p* < 0.05 compared to the original control (Wilcoxon signed-rank test). # *p* < 0.001 compared between the two groups (Mann–Whitney test).

Extracellular ATPs have strong pro-nociceptive properties associated with the activation of P2X3 receptors in trigeminal nerve afferents [32,33]. We compared the electrical activity of trigeminal afferents induced by ATP application in the control and pHHCY groups. In the control group, the baseline frequency of APs was 316.1 ± 24.4 APs per 5 min (n = 10; Figure 2a,b). ATP (100 μM) during the first 5 min of the application increased the number of APs up to 692.7 ± 38.9 per 5 min (n = 10; *p* = 0.005; Wilcoxon signed-rank test, Figure 2a,b). At the end of the application period, the AP frequency achieved the initial levels and was 394.4 ± 58.8 APs per 5 min (Figure 2b). During the incubation period of 20 min, the whole number of APs was 1207.4 ± 89.1 before and 2056.2 ± 186.5 APs during ATP application (n = 10; *p* = 0.005; Figure 2c).

In the pHHCY group, the initial AP frequency was higher compared to the control group, at 784.9 ± 128.6 in 5 min (n = 10; Mann–Whitney, *p* = 0.025; Figure 2b). ATP during the first 5 min of application significantly increased the firing frequency up to 1687.2 ± 169.9 APs in 5 min (n = 10; Wilcoxon signed-rank test, *p* = 0.005; Figure 2b). At the end of the application period, the AP frequency was still higher compared to the baseline level (1323.5 ± 174.2 APs per min) but decreased to initial values only after 10 min of the washout process (810.4 ± 132.5 APs per 5 min, Figure 2b).

During the incubation period of 20 min, the number of APs was 3005.0 ± 470.1 per 20 min before and 5793.3 ± 664.7 during ATP application (n = 10; Wilcoxon signed-rank test, *p* = 0.005; Figure 2c). The increase in the trigeminal nerve firing rate was higher in the pHHCY group (Mann–Whitney, *p* = 4.39639 × 10^−4^).

### 3.4. Ca^2+^ Transients in Isolated Cultured Cells of the TG of Rats with pHHCY

**Figure 3:**
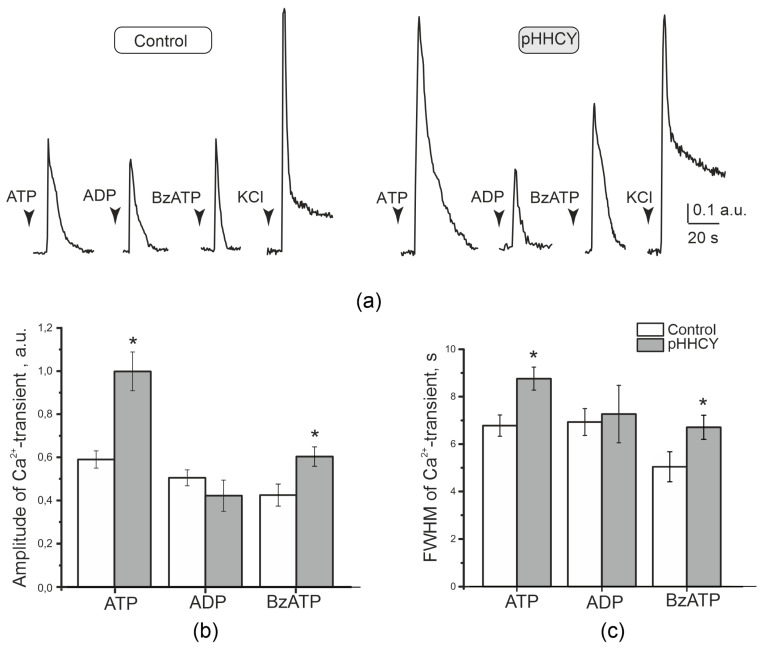
Ca^2+^ transients in TG neurons evoked by the application of purinergic receptor agonists. (**a**) Examples of Ca^2+^ transients in TG neurons in response to the application of ATP (100 μM), ADP (100 μM), and BzATP (30 μM) in the control and pHHCY groups. (**b**) The mean amplitude and (**c**) mean duration of Ca^2+^ transients in TG neurons in response to the application of ATP (100 μM), ADP (100 μM), and BzATP (30 μM) in the controls and in the conditions of pHHCY. ΔF = F − F_0_, where F is the peak fluorescence of cells and F0 is the background fluorescence close to a given cell. For statistical analysis, the amplitude of each agonist-induced transient was normalized using the amplitude of the KCl-induced Ca^2+^ transient for the same cell. * *p* < 0.05, Mann–Whitney U-test.

To characterize the functional properties of a large number of cells, we performed intracellular Ca^2+^ imaging of neurons and satellite glial cells (SGCs) activated by the agonists of the P2X and P2Y receptors. ATP (100 μM) was used as a non-selective agonist of the P2X and P2Y receptors, ADP (100 μM) as the selective agonist of metabotropic receptors, and BzATP (30 μM) as a partial agonist of ionotropic P2X receptors and as a relatively selective agonist of P2X7 receptors. Neurons were identified by their fluorescence response to KCl application (50 mM; 1 s). The application of ATP (2 s) caused Ca^2+^ transients in neuronal and satellite cells (Figure 3).

Generally, 54% of TG neurons of rats from the control group responded to ATP, while 57% of TG neurons in the pHHCY group were classified as ATP-responsive (Chi-square = 0.6, *p* > 0.05). In the control group, the amplitude of ATP-induced Ca^2+^ transients was evaluated as 0.59 ± 0.04 a.u., and the mean duration was 6.78 ± 0.45 s (n = 3, 129 cells). In the pHHCY group, the amplitude and duration of Ca^2+^ transients were higher at 0.99 ± 0.09 a.u. (n = 4, 205 cells, *p* = 0.00109, Mann–Whitney test, Figure 3a,b) and 8.76 ± 0.48 s (n = 4, 205 cells, *p* = 0.00802, Mann–Whitney U-test, Figure 3a,c), respectively.

The agonist of metabotropic P2Y receptors, ADP (100 μM), caused responses in 53% of TG neurons in control rats and in 48% of TG neurons of rats from the pHHCY group (Chi-square = 0.35, *p* > 0.05). The parameters of the ADP-induced Ca^2+^ transient did not significantly differ in the control and pHHCY groups. The amplitude was 0.5 ± 0.04 a.u. in the control group (n = 3, 85 cells) and 0.42 ± 0.07 a.u. in the pHHCY group (n = 4, 28 cells, *p* = 0.49803, Mann–Whitney, Figure 3a,b). The duration of Ca^2+^ transients was 6.9 ± 0.57 s (n =3, 85 cells) in the control group and 7.26 ± 1.21 s in the pHHCY group (n = 4, 30 cells, *p* = 0.89612, Mann–Whitney, Figure 3a,c).

The application of the partial agonist of ionotropic receptors BzATP (30 μM) caused Ca^2+^ transients in 41% and 45% of TG neurons of the control and pHHCY groups, respectively. The amplitude of Ca^2+^ transients in the control group was 0.42 ± 0.05 a.u. (n = 3, 30 cells) and 0.60 ± 0.04 a.u. in the pHHCY group (n = 4, 77 cells, *p* = 0.02, Mann–Whitney test, Figure 3b). The duration of the Ca^2+^ transients in the control group was 5.03 ± 0.63 s (n = 3, 30 cells), while in the pHHCY group, it was 6.71 ± 0.50 s (n = 4, 77 cells, *p* = 0.03, Mann–Whitney, Figure 3c).

**Figure 4:**
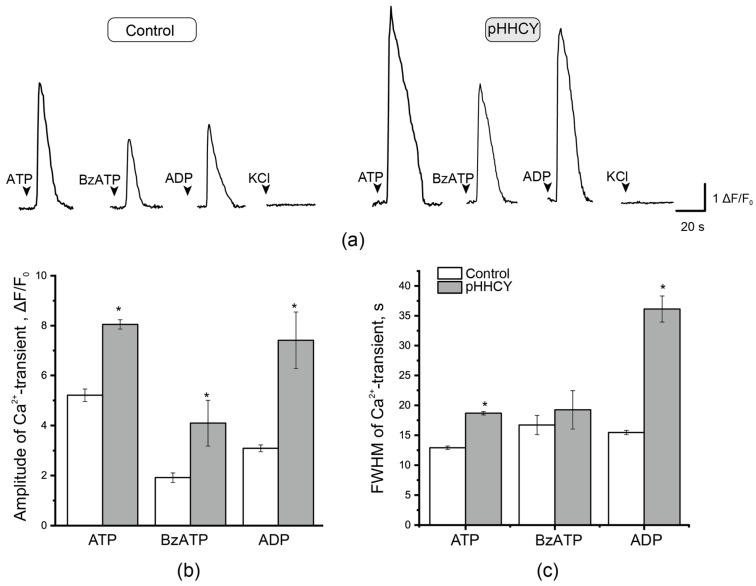
Ca^2+^- transients in the satellite glial cells of the trigeminal ganglion. (**a**) Original traces of fluorescent signals induced by the application of ATP (100 μM), ADP (100 μM), and BzATP (30 μM) in SGCs from the TG of rats from the control and pHHCY groups. The amplitude (**b**) and FWHM (**c**) of Ca^2+^ transients induced by the application of ATP (100 μM), ADP (100 μM), and BzATP (30 μM) in SGCs of rats from the control group (white column) and the pHHCY group (gray column). ΔF = F − F_0_, where F is the peak fluorescence of cells and F0 is the background fluorescence close to a given cell. * *p* < 0.05, Mann–Whitney U-test.

Since the SGCs of TG create a microenvironment for TG neurons and are actively involved in signaling cascades associated with the processes of inflammation and nociception, the parameters of Ca^2+^ transients of SGCs were analyzed. The amplitude and duration of Ca^2+^ transients of SGCs induced by the application of ATP, ADP, and BzATP were larger in the pHHCY group (Figure 4). The application of ATP (100 μM) induced Ca^2+^ transients in the SGCs of control animals, with an amplitude of 5.21 ± 0.25 ΔF/F_0_ (n = 3, 319 cells) and a mean duration of 12.91 ± 0.30 s (n = 3, 313 cells). In conditions of pHHCY, the amplitude increased to 8.05 ± 0.19 ΔF/F_0_ (n = 4, 680 cells, *p*= 2.31536 × 10^−22^, Mann–Whitney), and the duration raised to 18.67 ± 0.27 s (n = 4, 681 cells, *p* = 7.31947 × 10^−36^, Mann–Whitney). In the control group, the amplitude of ADP (100 μM)-induced responses was 3.09 ± 0.14 ΔF/F_0_ (n = 3, 207 cells), and the mean duration was 15.45 ± 0.35 s (n = 3, 199 cells). In the pHHCY group, both parameters were increased: the amplitude was 7.41 ± 1.13 ΔF/F_0_ (n = 3, 45 cells, *p*= 0.00218, Mann–Whitney), and the duration was 36.12 ± 2.18 s (n = 4, 46 cells, *p* = 0, Mann–Whitney).

The application of the relatively selective P2X7 receptor agonist, BzATP, induced Ca^2+^ transients in SGCs, with a mean amplitude of 1.91 ± 0.19 a.u. (n = 3, cells 45) in the control group and 4.09 ± 0.91 a.u. (n= 4, 101 cells, *p* = 0.00481, Mann–Whitney; Figure 4a,b) in the pHHCY group. The duration of Ca^2+^ transients was not significantly different: 16.71 ± 1.60 s and 19.24 ± 3.21 s for the TG neurons of the control rats and for animals from the pHHCY group, respectively (*p* > 0.05, Mann–Whitney; Figure 4a,c).

### 3.5. Ca^2+^ Transients in TG Neurons After Incubation in L-Homocysteine for 24 h

**Figure 5:**
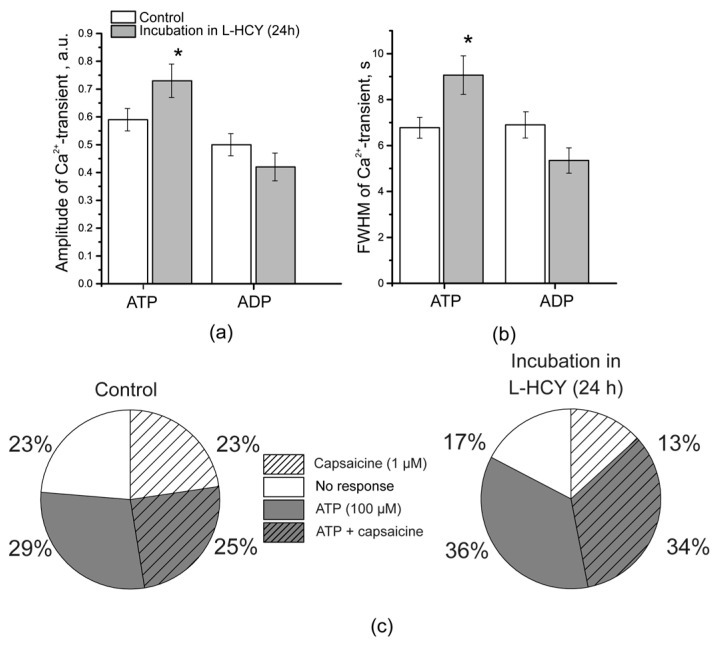
Ca^2+^ transients of TG neurons after 24 h of incubation in L-homocysteine. The amplitude (**a**) and duration (FWHM) (**b**) of Ca^2+^ transients in response to the application of ATP (100 μM) and ADP (100 μM) in TG neurons from the control group (white column) and after 24 h of incubation in L-homocysteine (L-HCY, 100 μM, gray column). Mean ± SEM. * *p* < 0.05, Mann–Whitney; (**c**) the percentage of TG neurons responding to ATP (100 μM, 2 s) and capsaicin (1 μM, 2 s) in the controls and after 24 h of incubation in L-HCY (100 μM). In the control group, 56 cells responded to ATP only; 44 responded to capsaicin only, and 48 responded to both ATP and capsaicin (194 cells). After 24 h of incubation in L-HCY, 33 cells responded to ATP only; 13 cells responded to capsaicin only, and 35 responded both to ATP and capsaicin (98 cells).

The incubation of the cultured TG neurons in the medium containing L-homocysteine (100 μM) for 24 h resulted in an increase in ATP-induced Ca^2+^ responses in neurons: the amplitude raised from 0.59 ± 0.04 a.u. in the controls to 0.73 ± 0.06 a.u. after incubation in L-homocysteine (n = 3, 65 cells, *p* = 0.04, Mann–Whitney), and a signal duration change from 6.78 ± 0.45 s in the controls to 9.07 ± 0.84 s after incubation in L-homocysteine was observed (n = 3, 61 cells, *p* = 0.01, Mann–Whitney; Figure 5a,b).

The amplitude and duration of Ca^2+^ transients induced by ADP (100 μM), however, did not change after incubation in L-homocysteine. After incubation, the amplitude of the ADP-induced Ca^2+^ transient was 0.42 ± 0.05 a.u. (*p* > 0.05, Mann–Whitney), with a mean duration of 5.35 ± 0.55 s (*p* > 0.05, Mann–Whitney, Figure 5a,b).

At the same time, 24 h of incubation in L-homocysteine increased the percentage of TG neurons responding simultaneously to ATP and capsaicin: from 25% in the controls to 34% after incubation (Chi-square = 3.85, *p* < 0.05, Chi-square test) (Figure 5c). Since TRPV1 receptors are mainly localized in small-diameter neurons and their presence distinguishes nociceptive neurons from other types of cells, an increase in the number of cells responding simultaneously to the TRPV1 receptor agonists, capsaicin and ATP, may identify an increased expression or activation of P2X receptors specifically in nociceptive neurons.

### 3.6. Mast Cell Degranulation in Response to BzATP in Rats with pHHCY

**Figure 6:**
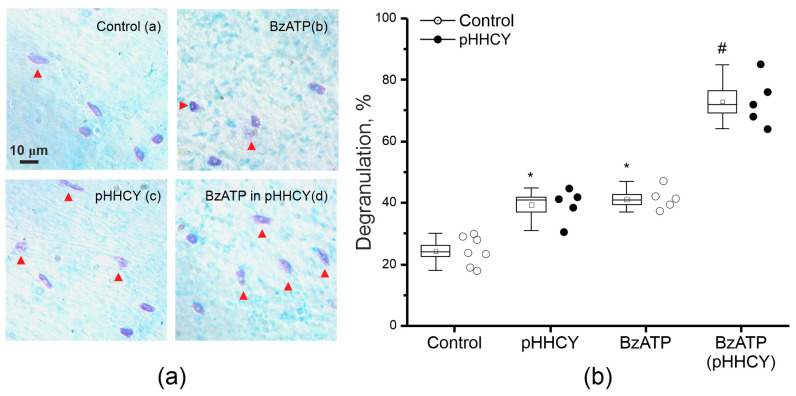
Mast cell degranulation in the dura mater of the control and pHHCY rats. (**a**) Original images of mast cells stained with toluidine blue in the control and pHHCY groups; mast cells of the control and pHHCY (rats after incubation in the solution containing BzATP (30 μM)) rats. The scale bar equals 10 μm; (**b**) the percentage of degranulated mast cells in the control and pHHCY groups * *p* < 0.05 compared to control values. # *p* < 0.05 between groups. Mann–Whitney test.

In the intact dura mater of the control rats, 24 ± 2% (n = 7) of the mast cells were degranulated (Figure 6a(a),b). The number of degranulated mast cells was significantly higher in the pHHCY group, and the mean percentage was 49 ± 3% (n = 5; Mann–Whitney *p* = 0.005; Figure 6a(c),b). The application of BzATP (a relatively selective agonist of P2X7 receptors, 30 μM) increased the fraction of degranulated mast cells in the dura mater of control animals up to 44 ± 3.2% (n = 5, Mann–Whitney *p* = 0.005; Figure 6a(b),b). Nevertheless, BzATP showed an even more pronounced effect in the conditions of pHHCY and increased the level of mast cell degranulation to 73 ± 3.6% (n = 5, Mann–Whitney *p* = 0.012; Figure 6a(d),b).

## 4. Discussion

Despite intensive studies of migraine mechanisms, the diversity of factors causing migraines does not allow for unifying approaches to treatment, and medication can cause side effects. At the same time, there are factors that increase the predisposition to migraine, and the elimination of these factors can be a preventative measure for the occurrence or worsening of the disease. One biomarker of migraine is homocysteine, which is elevated in the blood plasma or cerebrospinal fluid of patients, and it has been shown to increase the frequency or/and severity of migraine attacks [22,25]. However, the mechanisms of sensitizations of peripheral afferents of the trigeminal nerve innervating the meninges have not been identified. In the present study, we investigated the role of purinergic mechanisms in increasing the excitability of trigeminal afferents in rats with pHHCY.

Homocysteine levels in cells are carefully controlled due to the reactions of demethylation and transulfuration [3,4]. However, several conditions such as genetic mutations of *CBS* or polymorphism of *MTHFR,* vitamin B group deficiency, aging, and certain medication can increase the homocysteine levels of the blood plasma [3,4]. In clinical studies, higher homocysteine levels in plasma were found in patients with migraines with aura; in the cerebrospinal fluid, elevated homocysteine levels were shown for both groups, without and with aura [18,63,64].

In our animals, HHCY was modeled in female rats using a diet with a high methionine content. The offspring showed elevated plasma levels of homocysteine throughout the life along with several impairments in cognitive and motor behavior and muscle strength, and higher susceptibility was found in the experimental models of migraines with and without aura [14,17,23,24,51]. Indeed, lower concentrations of KCl triggered cortical spreading depression (CSD), and a higher number of CSD cases were generated during long-term KCl application in rats with pHHCY [17,23]. Homocysteine and its derivatives may act as excitatory agonists of NMDA glutamate receptors, which are important for CSD [65,66,67,68], and the upregulation of NMDA receptors may be involved in the central effects of pHHCY [69].

In the migraine model induced by nitroglycerine injections, rats with pHHCY were more sensitive to the injections of nitroglycerine [24], which indicated the sensitization of trigeminal nerve afferents of rats with pHHCY. At the level of meningeal afferents, lower concentrations of KCl induced an increase in the frequency of APs of the TG nerve, and lower currents were necessary to induce APs in TG neurons, indicating a higher excitability of TG neurons of rats with pHHCY [55]. This finding was further supported in the present work where 4-AP as an excitatory substance was used to assess tissue excitability. 4-AP is an inhibitor of voltage-gated transient fast-inactivating channels; K_A_ [70] is widely expressed in TG neurons and peripheral nociceptive afferents. IA currents contribute to maintaining the AP generation threshold in neurons, thereby influencing their excitability [71,72]. The inactivation of K^+^-type A channels can contribute to hypersensitivity in sensory neurons and increase the frequency of AP generation and decrease the AP duration [70,72,73,74]. The application of 4-AP at the area of innervation of the middle meningeal artery leads to the activation of Aδ- and C-fibers of the trigeminal nerve. In rats with pHHCY, lower concentrations of 4-AP (10 μM) were necessary to increase the AP frequency compared to control animals, where the frequency of AP, which increases only at a concentration of 25 μM, indicated the higher excitability of TG afferents. Moreover, we cannot exclude the suppression of IA currents in trigeminal afferents of rats with pHHCY; this result is similar to previous studies, where Kv depression was shown in neuropathic/inflammatory conditions [75].

NMDA receptors were shown to be targets of homocysteine action in various cells including TG neurons [65,76,77,78,79,80], and a previous study demonstrated that the acute application of homocysteine or its derivatives induced spiking of the TG nerve through the activation of NMDA receptors [55]. However, in rats with pHHCY, we did not find increased responses of TG neurons or trigeminal afferents to NMDA application [55].

P2X3 receptors are highly expressed in trigeminal sensory neurons [36] and provide ATP-induced activation of the trigeminal afferents to generate nociceptive signaling [31,39,81]. Previous data demonstrated that after nerve injury (or inflammation), various stimuli, including extracellular ATP, can more easily activate nociceptive neurons [82], which enhances the generation of APs propagating to the spinal cord and brain, and be perceived as pain signals. Indeed, in rats with pHHCY exogenous application of ATP, the receptive area of the peripheral afferents induced a more significant increase in the AP frequency compared to the controls, which suggests a higher sensitivity of the P2X3 and/or P2X2/3 receptors that are expressed in nociceptive Aδ-fibers and in C-fibers [32,83,84]. The expression and activity of ATP receptors, including ionotropic P2X2/X3 receptors, can be increased under inflammatory conditions [39], which was observed in HHCY conditions. Increased levels of cytokines like IL-1β, TNF-α, and IL-6 and oxidative stress markers were demonstrated in HHCY in rats and mice [43,85,86,87]. Additionally, a significant increase in blood–brain barrier permeability closely related to neuroinflammation was shown in different models of HHCY induced in adult mice or rats by single or chronic injections of homocysteine and pHHCY and in C57Bl/6 mice with heterozygous deletion of *CBS* [43,88,89,90]. The sensitization of the peripheral afferents results in a pathological decrease in the AP threshold of neurons and an increase in their firing rate [55]. Furthermore, the slower decline of the ATP response in trigeminal afferents in pHHCY rats proposes a modification of the desensitization properties of P2X3 receptors, further promoting sensitization [81]. Therefore, a decrease in the activation threshold of neurons can be accompanied by a change in the structure and function of P2X3 receptors [91].

This hypothesis was supported by Ca^2+^ imaging analysis of a large population of cells isolated from TG to the agonists of ionotropic and metabotropic purine receptors, including ATP, ADP, and BzATP [92]. It was found that the amplitude of Ca^2+^ transients in response to ATP and BzATP was larger in TG neurons of rats with pHHCY along with the higher percentage of TG neurons responding to purine receptor agonists. The activation of P2X receptors permeable to Ca^2+^ ions induces inward depolarizing currents in addition to Ca^2+^ transients [58]. Therefore, the amplitude of Ca^2+^ transients indirectly corresponds to the rate of depolarization that can attain the threshold level for AP generation [31]. These data indicate the upregulation of P2X receptors in TG neurons, whereas metabotropic receptor activity did not change. Thus, it is most likely that the increase in excitability of rat TG neurons is mainly caused by modulating the functional activity of ionotropic P2X receptors.

TG neurons are surrounded by SGCs, which, along with neurons, express CGRP glutamate; purinergic receptors can release a variety of signaling molecules like ATP, NO, cytokins, or neurotrophins, which may provide a cross-talk between neurons and glial cells [93]. In addition P2Y satellite glial cells express P2X7 receptors, whose activation leads to a massive release of ATP, which in turn enhances Ca^2+^ signaling in neurons [94,95]. In our experiments, SGCs exhibit increased Ca^2+^ responses to ATP, ADP, and BzATP, supporting our suggestion about the up-regulation of purinergic receptors in pHHCY. Similarly, a bidirectional signaling between neurons and glia cells via ATP was enhanced in Ca(v)2.1 α1 R192Q mutant knock-in mice as a model of familial hemiplegic migraine type 1 [96]. Long-term incubation (24 h) of TG neurons in a medium containing homocysteine showed an increase in Ca^2+^ signaling in neurons, and the effects were comparable to pHHCY. In addition, we found that more cells responded simultaneously to capsaicin and ATP, which indicates a sensitization of small nociceptive neurons expressing TRPV1, which colocalizes with CGRP in most of the TG neurons [97]. When P2X receptors are activated, the trigeminal nerve releases CGRP, which, in turn, has an algogenic and sensitizing effect on P2X3 receptors [81,98,99].

CGRP released form TG neurons can directly initiate the cAMP signaling cascade that activates the gene expression of purinergic (P2X3) receptor channels in neurons and purinergic (P2Y) receptors in SGCs [39,100]. This will further promote the depolarization of trigeminal afferents and the transmission of nociceptive stimuli [101]. Several studies revealed higher blood levels of CGRP levels during migraine attacks and in interictal periods in cases of chronic migraine [102,103,104]. Similarly, in pHHCY rats, plasma CGRP levels were higher, and these rats demonstrated basal allodynia and photophobia along with a higher sensitivity in a nitroglycerine-induced migraine model [23,24]. An increased CGRP level along with high levels of mast cell degranulation suggest neurogenic inflammation in the meninges of rats with pHHCY [55]. Meningeal mast cells are key component of neurogenic inflammation, and when activated, they release a variety of proinflammatory and pro-nociceptive mediators including cytokins, ATP, prostaglandins, and serotonin, which further contribute to migraine pain [33,105,106]. Mast cells of rats with pHHCY were more sensitive to the relatively selective agonist of P2X7 receptors, which is consistent with data obtained from murine macrophages where homocysteine increased IL-1β synthesis via the enhancement of P2X7 expression and NF-ĸB and ERK activation [48].

Future studies aimed at analyzing the expression level of the P2X3 receptor or the effects of CGRP inhibitors on nerve excitability in HHCY will help to identify a causal link between homocysteine and the upregulation of the purinergic system.

## 5. Conclusions

The results of our study propose that the increased excitability of the TG system during chronic HHCY results from the sensitization of TG neurons and their peripheral afferents due to increased activity of ionotropic P2X3/X2 receptors. Chronic HHCY induces oxidative stress and inflammation with an increased level of CGRP, which promotes the upregulation of P2X3/X2 receptors in TG neurons and P2X7 and P2Y receptors in the TG SGCs and mast cells of the dura mater. The degranulation of mast cells further aggravates proinflammatory conditions and the release of excitatory mediators like serotonin and ATP. Located on the nerve endings in peripheral tissues, P2X channels can drive the initial nerve impulse from the nociceptor receptive field. The upregulation of excitatory P2X3 and P2X2/3 ATP-gated channels directly sensitizes C-fibers via membrane depolarization and calcium entry to facilitate pain transmission. Sensory neuron sensitization may result in a decrease in the threshold for AP firing so that neurons may be more readily excited even by minimal stimuli [91]. Our findings reveal the mechanisms of the higher excitability of trigeminal meningeal afferents in rats with pHHCY and support clinical data indicating the high risk of migraine in patients with elevated plasma levels of homocysteine.

## Figures and Tables

**Table 1:** The effect of 4-AP on the electrical activity of the trigeminal nerve in the control and pHHCY rats.

**4-AP Concentration**	**Mean AP Frequency Per 20 min** **in the Control Group**		**Mean AP Frequency Per 20 min** **in the pHHCY Group**	
	**Baseline** **Values**	**4-AP **	**Baseline Values **	**4-AP **
10 μM	959.2 ± 207.6	1006.5 ± 189.3 (*p* = 0.58) n = 4	1282.5 ± 75.7	3813.1 ± 586.3 * (* *p* = 0.036) n = 6
25 μM	1445.1 ± 53.5	1526.2 ± 112.7 (*p* = 0.41) n = 5	1842.6 ± 134.9	5131.1 ± 715.9 * (* *p* = 0.04) n = 6
50 μM	939.6 ± 105.2	3469.1 ± 820.1 * (* *p* = 0.01) n = 8	775.8 ± 45.2	5412.5 ± 461.4 * (* *p* = 0.04) n = 5
100 μM	611.2 ± 123.9	2460.6 ± 771.5 * (* *p* = 0.036) n = 6	943.6 ± 41.8	6600.8 ± 886.8 * (* *p* = 0.036) n = 6

* *p* < 0.05 compared the AP frequency before the 4-AP application (Wilcoxon signed-rank test).

## Data Availability

The data used to support the findings of this study are available from the corresponding author upon request.

## Abbreviations

The following abbreviations are used in this manuscript:

HHCY	Hyperhomocysteinemia
pHHCY	Prenatal HHCY
ATP	Adenosine triphosphate
ADP	Adenosine diphosphate
BzATP	2′(3′)-O-(4-Benzoylbenzoyl)adenosine-5′-triphosphate tri(triethylammonium) salt
TG	Trigeminal ganglion
CGRP	Calcitonin gene-related peptide
AP	Action potential
SGCs	Sattelite glial cells
4-AP	4-aminopiridine
CSD	Cortical spreading depression
MTHFR	5,10-methylenetetrahydrofolate reductase
FWHM	Full width at half maximum
